# A ligation and restriction enzyme independent cloning technique: an alternative to conventional methods for cloning hard-to-clone gene segments in the influenza reverse genetics system

**DOI:** 10.1186/s12985-020-01358-2

**Published:** 2020-06-23

**Authors:** Sushant Bhat, Dagmara Bialy, Joshua E. Sealy, Jean-Remy Sadeyen, Pengxiang Chang, Munir Iqbal

**Affiliations:** The Pirbright Institute, Surrey, UK

**Keywords:** Influenza, Reverse genetics, Polymerase, Restriction enzyme independent cloning

## Abstract

**Background:**

Reverse genetics is used in many laboratories around the world and enables the creation of tailor-made influenza viruses with a desired genotype or phenotype. However, the process is not flawless, and difficulties remain during cloning of influenza gene segments into reverse genetics vectors (pHW2000, pHH21, pCAGGS). Reverse genetics begins with making cDNA copies of influenza gene segments and cloning them into bi-directional (pHW2000) or uni-directional plasmids (pHH21, pCAGGS) followed by transfection of the recombinant plasmid(s) to HEK-293 T or any other suitable cells which are permissive to transfection. However, the presence of internal restriction sites in the gene segments of many field isolates of avian influenza viruses makes the cloning process difficult, if employing conventional methods. Further, the genetic instability of influenza gene-containing plasmids in bacteria (especially Polymerase Basic 2 and Polymerase Basic 1 genes; PB2 and PB1) also leads to erroneous incorporation of bacterial genomic sequences into the influenza gene of interest.

**Methods:**

Herein, we report an easy and efficient ligation and restriction enzyme independent (LREI) cloning method for cloning influenza gene segments into pHW2000 vector. The method involves amplification of megaprimers followed by PCR amplification of megaprimers using a bait plasmid, *DpnI* digestion and transformation.

**Results:**

Hard-to-clone genes: PB2 of A/chicken/Bangladesh/23527/2014 (H9N2) and PB1 of A/chicken/Bangladesh/23527/2014 (H9N2), A/chicken/Jiangxi/02.05YGYXG023-P/2015 (H5N6) and A/Chicken/Vietnam/H7F-14-BN4–315/2014 (H9N2) were cloned into pHW2000 using our LREI method and recombinant viruses were subsequently rescued.

**Conclusion:**

The LREI cloning procedure represents an alternative strategy for cloning influenza gene segments which have internal restriction sites for the enzymes used in reverse genetics. Further, the problem of genetic instability in bacteria can be alleviated by growing recombinant bacterial cultures at a lower temperature. This technique can be applied to clone any influenza gene segment using universal primers, which would help in rapid generation of influenza viruses and facilitate influenza research and vaccine development.

## Background

Reverse Genetics (RG) is the process of in vitro generation of live virus with synthetic or PCR amplified genes [1]. This technique enables the creation of mutant influenza viruses of any desired genotype or phenotype. The RG technique was first employed for the rabies virus in the year 1994 [2], and this was soon followed by the establishment of the in vitro generation of a range of DNA and RNA viruses (including segmented or non-segmented RNA viruses) [3–15]. The RG technology has also revolutionized the influenza field, progressing influenza research by way of genetically engineered recombinant influenza viruses. Reverse genetics as a tool has helped in studying the influenza host range [16, 17], transmission patterns [18] viral genome replication, pathogenicity and virulence [19–21]. This technique has also been implemented to develop influenza vaccines [22, 23] or recombinant influenza viruses harbouring reporter genes for studying virus egress and dissemination [24].

Despite the utility of RG systems, the cloning step remains a limiting factor for the *de-novo* generation of viruses. Gene cloning is a crucial step in RG technology and has gained popularity in terms of usage but the technique involves restriction digest [25] followed by ligation, which sometimes becomes difficult to perform. The primary reasons are presence of internal restriction enzyme sites (eg. for *BsmBI*, *BsaI, AarI* or *BbsI*) in the different gene segments of field isolates of influenza virus. Further, the degradation of dNTPs in the ligation buffer or inefficient ligase enzyme also result in failure during ligation. The RG plasmids harbouring large inserts (> 2000 bp) of influenza virus gene segments have also been shown to be unstable after transformation into *E. coli* cells [26, 27], which may be due to their toxicity to the bacterial host [28, 29]. This leads to incorporation of bacterial sequences into the target insert. As such, an alternative strategy for cloning is sought after. Ideally it would bypass the restriction-ligation steps, increase the efficacy of recombinant plasmid formation and reduce the chances of genetic recombination in the insert. Taking these aims into consideration, we have developed a ligation and restriction enzyme independent (LREI) cloning procedure for cloning influenza gene segments into the standard reverse genetics pHW2000 plasmid [30]. LREI cloning increases the chances of recombinant plasmid formation which if followed by growing bacteria at lower temperatures alleviates the problem of genetic recombination. Our work would be particularly beneficial to researchers who utilise the pHW2000 plasmid in RG workflows with influenza virus genes.

## Materials and methods

### Primer design

Primers were designed in such a way that the 5′ end of the forward primer contains 16 nucleotides complementary to the one end of pHW2000 multiple cloning site (MCS) followed by 12 conserved nucleotide bases of influenza untranslated region (UTR) and 2–6 segment specific nucleotides towards the 3′ end. The reverse primer constitutes 13 bases complementary to the other end of the pHW2000 MCS followed by influenza UTR, which contains the conserved 13 bases followed by 5–6 segment specific bases at the 3′ end. The primer sequences for cloning PB2 and PB1 are described in Table 1. The accession numbers of the polymerase genes used in this study are described in Table 2.

**Table 1:** Primers used for amplification of PB2 and PB1 gene segments and subsequent cloning into pHW2000 vector

Gene	Forward Primer (5′ to 3′)	Reverse Primer (5′ to 3′)	Expected Size (bp)
PB2	*TCCGAAGTTGGGGGGG***AGCGAAAGCAGG**TC	*CCGCCGGGTTATT***AGTAGAAACAAGG**TCGTTT	2370
PB1	*TCCGAAGTTGGGGGGG***AGCGAAAGCAGG**CAAAC	*CCGCCGGGTTATT***AGTAGAAACAAGG**CATTT	2370

The forward and reverse primers contain 16 and 13 nucleotides, respectively which are complementary to the pHW2000 (*italics)*, followed by 12 and 13 nucleotides forming conserved UTR (**bold**) and 2–6 gene specific nucleotides (underlined). The nucleotide sequences (*in italics*) anneal to the pHW2000 and allow directional cloning of desired gene segment into the pHW2000 vector

**Table 2:** Accession number (retrieved from GISAID/NCBI) of Polymerase genes used in the present study

Gene	Virus	Subtype	GISAID ID/ NCBI Accession Number	Blasts with *E. coli* type
PB2	A/chicken/Bangladesh/23527/2014	H9N2	KT362035	2012-EL-2448
PB1	A/chicken/Vietnam/H7F-14-BN4–315/2014	H9N2	EPI_ISL_327772	K12
	A/chicken/Bangladesh/23527/2014	H9N2	KT362036	K12
	A/chicken/Jiangxi/02.05YGYXG023-P/2015	H5N6	EPI_ISL_199401	K12

Nucleotide blast of PB2 and PB1 shows atleast 1% homology with bacterial genome

### Amplification of Megaprimer

A target amplicon (known as a megaprimer) with 5′ and 3′ overhangs complementary to the cloning site in pHW2000 plasmid which can facilitate the annealing of the megaprimer with the template plasmid was generated by PCR. The PB1 megaprimer of A/Chicken/Vietnam/H7F-14-BN4–315/2014/H9N2 [Vietnam/2014], A/chicken/Bangladesh/23527/2014/H9N2 [Bangladesh/2014], and A/chicken/Jiangxi/02.05YGYXG023-P/2015/H5N6 [Jiangxi/2015] viruses and PB2 megaprimer of Vietnam/2014 virus were amplified from the respective amplicons present in the pMKT or pMK-RQ cloning vector (GeneArt® -Thermo Fisher Scientific) using the primers mentioned in Table 1. The PB1 megaprimer was also amplified from the viral RNA of Vietnam/2014 virus. Briefly, the viral RNA was extracted using QIAamp Viral RNA Mini Kit (Qiagen) and reverse transcribed using Verso cDNA Synthesis Kit (ThermoFisher Scientific) and universal 12 primers (Uni 12) [31]. The PB1 megaprimer was amplified using 20 nM of forward and reverse primers (Table 1) and Pfu Ultra II hotstart PCR master mix (Agilent Technologies). The PCR amplification steps include: initial denaturation at 95 °C for 3 min, followed by 35 cycles of denaturation (95 °C for 30s), annealing (58 °C for 30 s) and extension (72 °C for 3 min) followed by final extension (72 °C for 5 min). The amplified virus gene-specific amplicons (Megaprimers) were purified using QIAquick gel extraction kit (Qiagen) and quantified using nanodrop (ThermoFisher Scientific).

### Cloning PCR, DpnI digestion and transformation

**Fig. 1:**
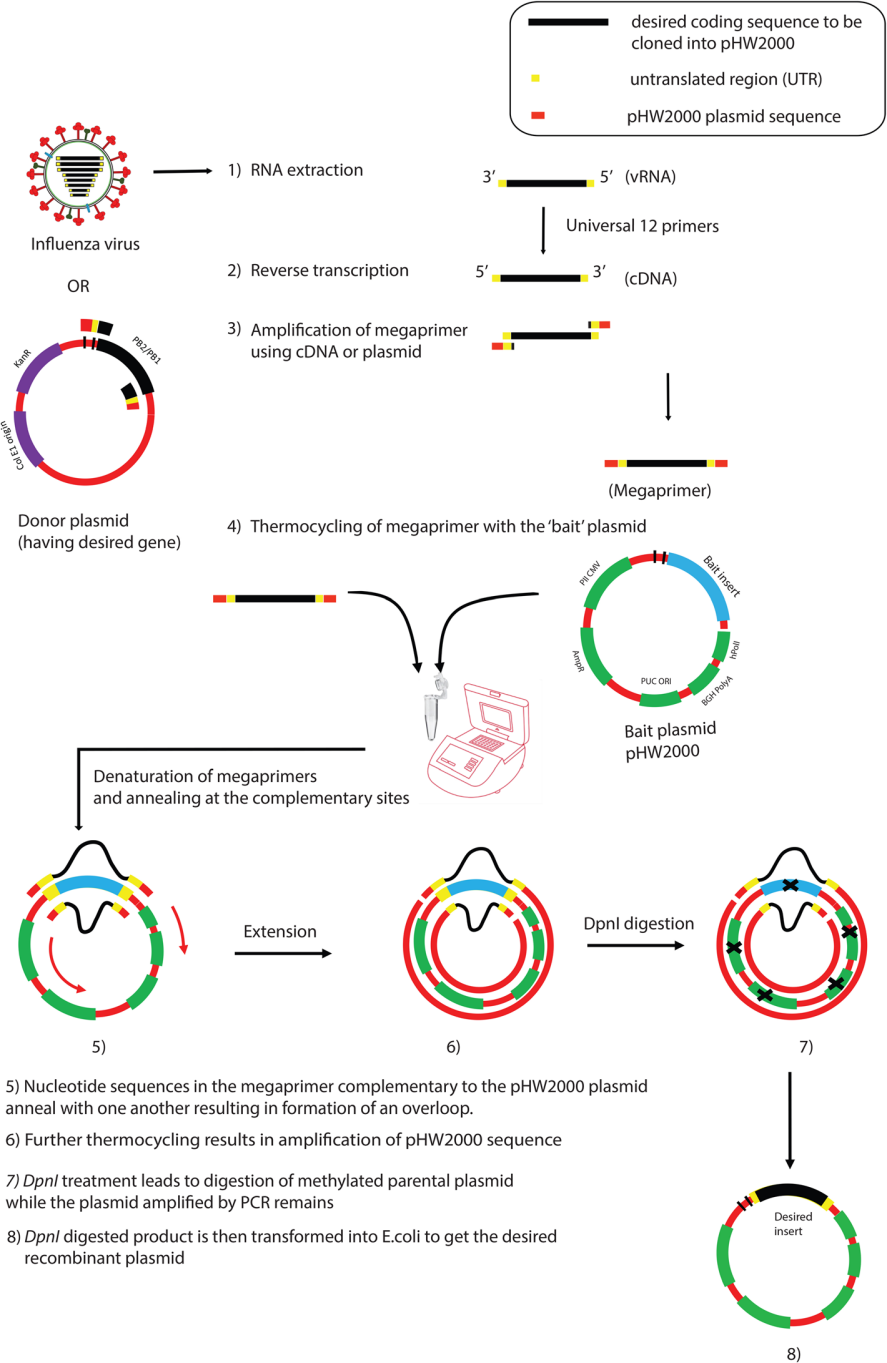
A schematic representation of the LREI cloning procedure. The technique involves designing primers that incorporate the gene specific untranslated region (UTR) (yellow) and nucleotides homologous to the plasmid pHW2000 (red) multiple cloning site (MCS) to the polymerase coding region resulting in formation of a megaprimer. The viral RNA extracted from the influenza virus (1) can be reverse transcribed by using universal 12 primers (AGCAAAAGCAGG) (2), and the megaprimer can be amplified either from cDNA or any donor plasmid using the primers mentioned in Table 1 (3). Denaturation of the megaprimer generates two primers having complementary ends to the pHW2000 MCS, which when used with a bait plasmid facilitate annealing (4,5). Subsequent thermocycling steps result in extension of the annealed primers (5) thereby synthesizing the non-methylated DNA insert along with the pHW2000 vector as a mixture. *DpnI* treatment results in digestion of parental methylated DNA, (7) leaving the newly synthesized non-methylated DNA, which can be transformed into *E. coli* resulting in formation of the desired recombinant plasmid (8)

Purified megaprimers (250 ng) were subjected to thermocycling using Pfu Ultra II hotstart PCR master mix (Agilent Technologies) using pHW2000 containing Polymerase Acidic (PA) gene from A/chicken/Bangladesh/23527/2014 (H9N2) [Bangladesh/2014] (100 ng) (Fig. 1). The thermocycling conditions include: initial denaturation at 95 °C for 4 min followed by 45 cycles of denaturation (95 °C for 30 s), annealing (55 °C for 30 s) and extension (72 °C for 6 min) followed by final extension at 72 °C for 10 min. *DpnI* digestion of the parental template was carried out by addition of 3 μl of *DpnI* to the reaction mix and incubated at 37 °C for 2 h. The reaction was stopped by heating at 80 °C for 10 min. 15 μl of the *DpnI* digested product was used for transformation of one shot DH5α cells (Invitrogen)/XL-Gold competent bacteria. The plates were incubated overnight at 32 °C. The PB2 and PB1 positive colonies of Bangladesh/2014 and Vietnam/2014 H9N2 were screened by plasmid PCR of the extracted plasmid using Hoffmann primers [31]. The PB1 positive colonies of Jiangxi/2015 were screened by colony PCR using the primers H5N6 PB1F (GAAGTTGGGGGGGAGCGAAAGCAGGC) and H5N6 PB1-R (CATCACATCCTTGAGGAAATCTATTAG) followed by confirmation by plasmid PCR using Hoffmann primers [31]. The desired recombinants were further confirmed by nucleotide sequencing using T7F and bGH R primers.

### Virus rescue

A standard influenza virus reverse genetics protocol was followed to rescue influenza viruses [1]. Briefly, 1 μg of recombinant DNA plasmids carrying Vietnam/2014 virus cDNAs were mixed with Lipofectamine 2000 (Life Technologies) and transfected into HEK-293 T cells. At 24 h post-transfection, the HEK-293 T cells were co-cultured with MDCK cells in the presence of TPCK treated trypsin (Sigma). After 72 h post co-culture, presence of rescued recombinant virus in culture supernatants was confirmed by standard Haemagglutination Assay [32] and Plaque Assay [33].

## Results

### PB2 and PB1 could not be cloned by conventional restriction digestion and ligation

The PB2 and PB1 genes of H5N6 and H9N2 viruses (Table 2) could not be cloned into pHW2000 by standard cloning procedures as mentioned by Hoffmann et al [30]. Either there were no colonies, or the 20–40 colonies screened were all negative for the desired insert.

### Ligation and restriction enzyme independent (LREI) cloning procedure using PA-pHW2000 as a bait plasmid improved the cloning efficiency

The ligation independent cloning PCR was initially performed by using megaprimers and empty pHW2000 or pHW2000 containing M gene as a bait plasmid. But this didn’t yield any positive colonies after *DpnI* digestion and transformation. Since, the three polymerase genes (PB2, PB1 and PA) contain same UTR sequences, thus, to improve the annealing efficiency of megaprimer with the bait plasmid, pHW2000 containing PA gene was taken as a bait plasmid (Fig. 1) and cloning PCR steps were performed. Transformation followed by growth of transformed bacteria at 37 °C resulted in colonies which showed PB2 and PB1 specific bands after screening with PB2 and PB1 specific primers. However, nucleotide sequencing of the plasmids showed some foreign gene insertions, which possibly were inserted by genetic recombination with the bacterial sequence.

### Growth of recombinant culture at 32 °C reduced bacterial recombination

**Fig. 2:**
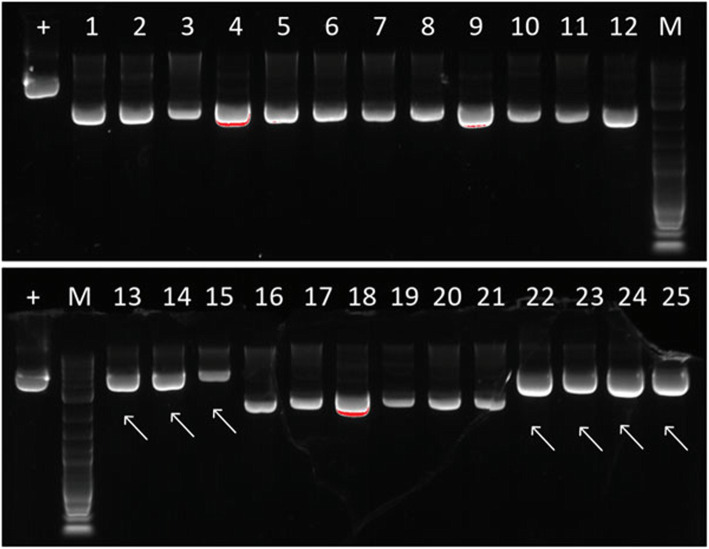
The PB1 positive colonies of Bangladesh/2014 and Vietnam/2014 H9N2 virus were screened by plasmid PCR using the PB1 primers [31]. Out of 16 colonies screened for Bangladesh PB1 (lane 1–16), 3 were positive (13, 14, 15; indicated by arrows) while for 9 colonies screened for Vietnam PB1 (lane 17–25), 4 were found to be positive (22, 23, 24, 25; indicated by arrows). The positive clones were further confirmed by nucleotide sequencing using T7F and BGHR primers. (+ = positive control; M = DNA marker)

**Fig. 3:**
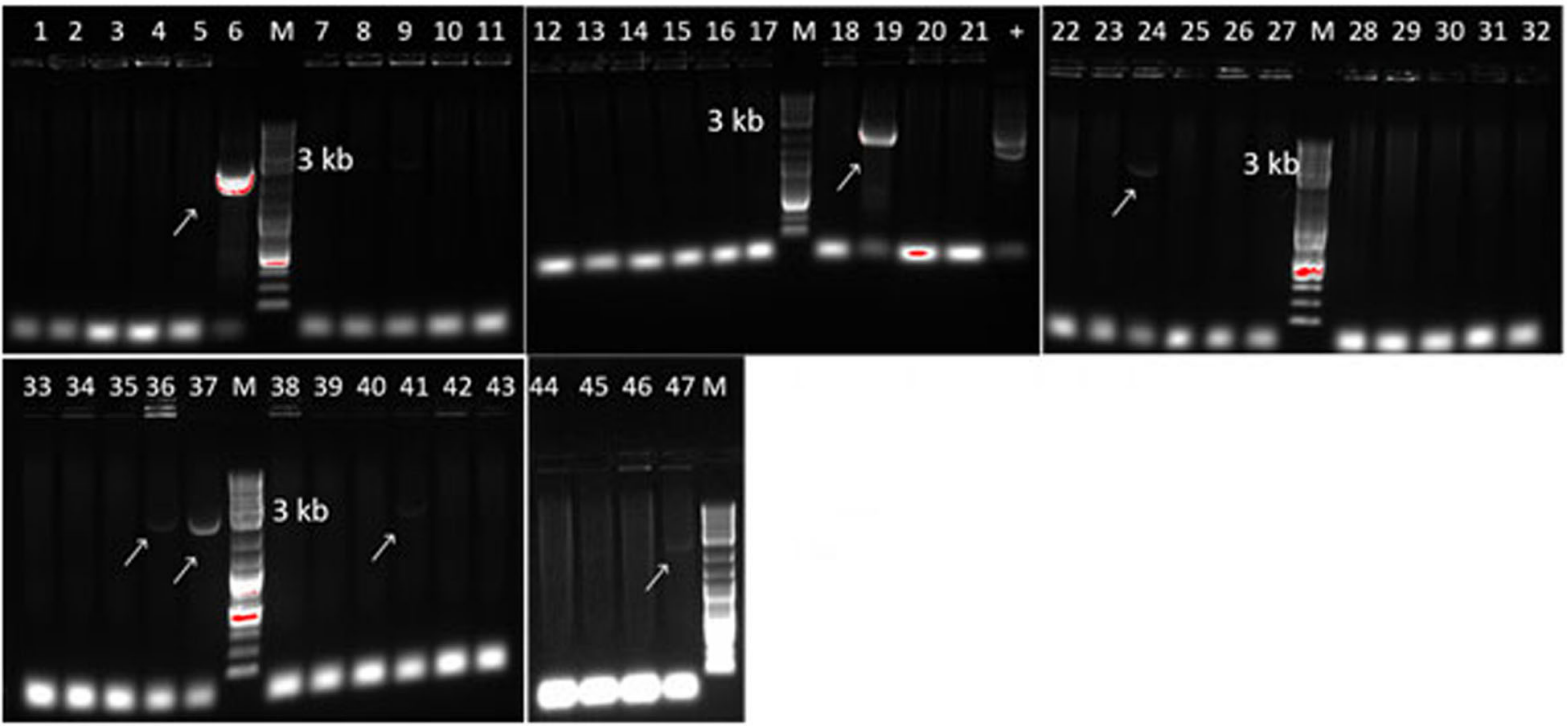
The PB2 positive colonies of Bangladesh/2014 H9N2 virus were screened by plasmid PCR using the PB2 primers [31]. Out of 47 colonies screened (lane 1–47) for Bangladesh/2014 PB2, 7 were positive in plasmid PCR (indicated by arrows). The positive clones were further confirmed by nucleotide sequencing using T7F and BGHR primers. (+ = positive control; M = DNA marker)

**Fig. 4:**
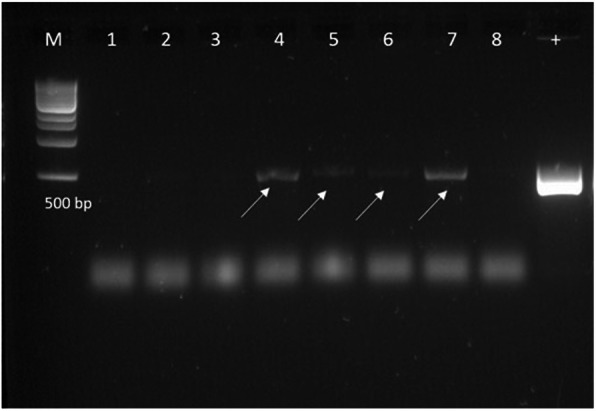
The PB1 colonies of Jiangxi/2015 were screened by colony PCR. A portion of 550 bp of PB1 was amplified using the primers H5N6 PB1F (GAAGTTGGGGGGGAGCGAAAGCAGGC) and H5N6 PB1-R (CATCACATCCTTGAGGAAATCTATTAG). Eight colonies were screened by colony PCR. The positive colonies in colony PCR (indicated by arrows) were further confirmed by plasmid PCR and nucleotide sequencing using T7F and BGHR primers (+ = positive control; M = DNA marker)

To reduce the bacterial recombination between the desired plasmid and bacterial genome, temperature for growth of the recombinant culture was reduced to 32 °C from 37 °C. Colony screening by plasmid PCR (Figs. 2, 3 and 4) showed PB2/PB1 specific colonies and further nucleotide sequencing showed desired PB2 and PB1 inserts without any recombination.

### LREI method saves time when cloning involves multiple gene segments

The LREI cloning method is faster than the conventional cloning methods due to bypassing the restriction digestion of the insert amplicons and plasmids and the ligation procedures.

### The cloned plasmids can efficiently be used to generate viruses de-novo

To confirm the functionality of the cloned genes, the plasmids were checked for their ability to rescue influenza virus in-vitro. All the polymerase plasmids were functional and reassortant viruses could be rescued by using the 8 plasmid reverse genetics system.

## Discussion

Here we developed a novel LREI directional cloning technique which bypasses the restriction digestion step and thus can be employed to increase the cloning efficiency of influenza gene segments having internal restriction sites into pHW2000 vector. The primers (Table 1) were designed to target conserved non-coding region (NCR) of influenza genes such that the approach can be used for targeted cloning of any gene of influenza A virus with the exception of few subtypes of Neuraminidase (NA) due to three nucleotide difference at the 3′ and 5′ ends in their NCR [31]. This technique used PCR to create a target amplicon known as a megaprimer with 5′ and 3′ overhangs. These overhangs are complementary to the cloning site in pHW2000 plasmid and facilitate the annealing of the megaprimer with the template plasmid. Thermocycling was conducted to anneal the megaprimer with pHW2000, which results in the formation of an overloop (Fig. 1). However, to minimise the chances of self-annealing of megaprimers and to increase the chances of annealing with the bait plasmid, a higher concentration of the bait plasmid can be used. *DpnI* digestion of the parental methylated DNA followed by transformation of the nicked-circular plasmid results in generation of desired recombinant plasmid without involving restriction digestion and DNA ligation. Furthermore, the unique overhang present in the megaprimer results in directional cloning of the desired insert. This was confirmed by Sanger sequencing of the cloned plasmids. The LREI cloning strategy allows for the integration of any gene into any site of the vector, provided the same strategy of megaprimer is followed.

The pHW2000 vector is a bidirectional plasmid that has RNA PolI and PolII promoters for the generation of influenza vRNA and mRNA. Thus, cloning a cDNA copy of all the gene segments into pHW2000 plasmid and transfection of recombinant plasmids into HEK-293 T cells results in de novo generation of influenza virus [30]. Cloning remains a critical factor for the rapid generation of influenza viruses in vitro. As a proof of principle for establishment of the LREI cloning procedure, we sub-cloned the PB2 gene of Bangladesh/2014 H9N2 and PB1 genes of Vietnam/2014 H9N2, Bangladesh/2014 H9N2, and Jiangxi/2015 H5N6 from pMKT/pMK-RQ vector (GeneArt®) into the pHW2000 vector. The technique involved choosing a bait plasmid that can assist in formation of a desired recombinant. In the first instance, using empty pHW2000 and pHW2000 vector containing Matrix (M) gene as bait plasmids didn’t result in any colonies being formed, possibly due to instability of the overloop (Fig. 1) [5]. We assumed the size difference between the desired insert and the insert present in the bait plasmid could be a key to generate the desired recombinant plasmid. Since the conserved nucleotides in the UTR of the Polymerase genes are similar, to minimise the size of the overloop formed during the thermocycling with the megaprimer and to maximize the possibility of generation of successful recombinant by cloning PCR, we used pHW2000 containing PA insert as a bait plasmid (Fig. 1 ) [4].

While doing LREI cloning, we experienced bacterial recombination in the desired gene, which normally occurs as an outcome of increased metabolic burden on recombinant bacteria, due to concatemer rich sequences in the insert [34, 35] secondary or tertiary structures in the DNA, too low or too high copy number of the plasmid [36], genotype of the competent cells [37, 38], length of the cloned segment or temperature used to grow the culture [39, 40]. Although the exact reason for experiencing difficulties in cloning is difficult to pin point, there seemed to be no convincing role of DNA secondary structures or GC content (data not shown). However, nucleotide blast showed that PB1 gene segments had around 1% sequence homology with the *E. coli* K-12 genome which is the progenitor of most of the commercially available lab strains of *E.coli*. Based on the published reports, sequence homology can contribute to homologous recombination leading to deletion/insertions in target insert [41]. Thus, as an effort to reduce the metabolic burden on the transformed bacteria, all the incubation steps involving growth of recombinant bacteria were performed at 32 °C instead of 37 °C. Nucleotide sequencing of all the plasmids for target gene inserts further confirmed the presence and sequence orientation of the desired gene and absence of transposable elements. The colonies that carried PB2 and PB1 gene segments were also found to be relatively smaller in size, compared to other colonies which were negative by PCR, suggesting that small colonies likely contain the plasmids that incorporate the correct length PB2 and PB1 gene insert in contrast to the larger size colonies which generally contained empty plasmid or a plasmid with shorter or truncated versions of the gene inserts [26]. This can potentially also be due to the metabolic burden on the recombinant bacteria, which could be associated with the plasmid DNA replication and which eventually leads to reduction in the growth rate of the recombinant bacterial cells [39]. However, growing the recombinant bacteria at 32 °C doesn’t necessarily prevent the insertion of bacterial sequences into the cloned influenza gene. Although we did not notice any recombination in the polymerase genes grown at 32 °C in the present study, we have encountered the problem of genetic recombination while doing site-directed mutagenesis of smaller segments like HA and NS even at 32 °C. This was countered by further reduction of temperatures to 30 °C or sometimes the recombinant cultures were incubated at room temperature. However, this reduces the bacterial growth in the recombinant culture and can affect the plasmid yield.

To confirm the efficacy of the proposed method, LREI cloning was also utilized to clone PB1 of Vietnam 2014 H9N2 by using megaprimer amplified from viral RNA using RT-PCR employing PB1 specific primers (Table 1).

Our LREI cloning procedure can efficiently be employed to clone influenza gene segments from field isolates having internal restriction sites for the standard enzymes used in reverse genetics system. The technique has been used to clone the Neuraminidase (NA) gene of a field isolate of H9N2 virus having internal restriction sites for *BsaI* [42] using pHW2000 containing M gene as a bait plasmid. Likewise, the bait plasmid containing M gene can be used to clone Haemagglutinin (HA) and Nucleoprotein (NP) genes into pHW2000. For cloning of smaller segments like M and Non-Structural (NS) genes, empty pHW2000 vector can be used as a bait plasmid. LREI cloning technique is also quicker and takes less than 2 days for cloning and confirmation of the desired clone (Table 3). Furthermore, the cloned cDNAs could efficiently generate influenza viruses de novo. Thus, our LREI technique is more robust and efficient for *de-novo* synthesis of influenza viruses and complements the 8 plasmid reverse genetics system.

**Table 3:** Comparison of relative time taken by LREI cloning compared to conventional cloning

Steps in cloning procedure		Time required during different cloning steps	
		Conventional cloning	Ligation and Restriction Enzyme Independent (LREI) Cloning^a^
a	Viral RNA extraction and cDNA synthesis	2.5 h	2.5 h
b	Generation of desired amplicon by thermocycling	3–5 h	3–5 h
c	Agarose gel electrophoresis and gel extraction of desired amplicon	2 h	2 h
d	Restriction digestion of desired amplicon and Restriction digestion of the cloning vector	1 h – 16 h (depending upon the enzyme used)	**Not required**
e	Purification and quantification of the digested amplicon and the cloning vector	1–2 h	
f	Quick ligation or overnight ligation of the digested amplicon and cloning vector	1 h – 16 h (depending upon the ligation kit)	
g	Transformation	1.5 h	1.5 h
h	Screening of positive colonies	3–7 h	3–7 h

^a^Our LREI cloning saves 2 days of time while cloning and is more efficient in cloning unstable polymerase genes into standard cloning vector

Various strategies for cloning have been reported, which include: TA cloning [43], GATEWAY recombinational cloning [44], CloneEZ one step cloning [45], and cloning by overlap extension PCR [46]. Each method has its own limitations e.g. TA cloning using standard Taq DNA Polymerase may result in point mutations in the amplicon during PCR amplification of the desired amplicon and further required specific sequences to create overhangs that would facilitate cloning procedures. Another technique called Gateway recombinational cloning requires DNA recombination to transfer DNA between donor and destination vectors, but this requires additional sequences for recombination. CloneEZ kits use sticky ends in the vector and insert for cloning but linearization of vector by restriction digestion is required.

Like LREI, another approach involving the use of *ccd*B gene as a selection marker has also been used to insert the influenza PB2 and PB1 genes into the pHWS*ccd*B vector [47]. Our approach neither requires any selection marker nor is there any requirement for modification of pHW2000 vector. Similarly, many novel approaches to molecular cloning including Homologous recombination [48, 49], PLICing [50] and use of Zinc finger nucleases [51] have been proposed in recent years that also don’t require restriction enzymes. Many researchers have reported similar strategies of DNA cloning by PCR which include restriction site-free cloning [52], restriction free cloning [53], cloning by overlap extension PCR [46] and MEGAWHOP cloning [54].

Our LREI cloning technique is based on exponential amplification of a megaprimer and the targeted vector, which results in a greater number of positive colonies after transformation compared to the conventional cloning strategies. This technique is specific and highly efficient in generation of cloned plasmids, which are otherwise difficult to clone. LREI cloning increases the chances of formation of recombinant clones and growth of recombinant bacteria at lower temperatures alleviates the problem of genetic recombination, albeit at the cost of plasmid yield. In summary, this technique can be applied to clone all influenza gene segments using universal primers, which would help in rapid generation of influenza viruses and make the study of influenza virus biology easier.

## Conclusion

The LREI cloning procedure represents an alternative strategy for cloning influenza gene segments which have internal restriction sites for the enzymes used in reverse genetics. Further, the problem of genetic instability in bacteria encountered in influenza gene segments can be alleviated by growing recombinant bacterial cultures at a lower temperature. This technique can be applied to clone any influenza gene segment using universal primers, which would help in rapid generation of influenza viruses and facilitate influenza research and vaccine development.

## Data Availability

The authors are happy to share any data or material upon request.

## Abbreviations

LREI: Ligation and Restriction Enzyme Independent
RG: Reverse Genetics
MCS: Multiple Cloning Site
UTR: Un-translated Region
PB2: Polymerase Basic 2
PB1: Polymerase Basic 1
PA: Polymerase Acidic
HA: Haemagglutinin
NP: Nucleoprotein
NA: Neuraminidase
M: Matrix
NS: Non-structural
